# Enhancement of volatile fatty acids production through anaerobic co-digestion of navel orange residue and waste activated sludge: Effect of pre-treatment and substrate proportions

**DOI:** 10.1016/j.heliyon.2023.e19777

**Published:** 2023-09-03

**Authors:** Shan-Yan Dong, Jin-Cai Luo, Gang Chen, Shuai Tian, Hong Sun, Xiang-Zhe Xiao, Yi-Chun Zhu

**Affiliations:** a School of Civil and Surveying & Mapping Engineering, Jiangxi University of Science and Technology, Ganzhou, 341000, China; b Jiangxi Province Ganzhou key laboratory of Basin pollution simulation and Control, Jiangxi University of Science and Technology, Ganzhou, 341000, China; c School of Resources Environmental Engineering, Jiangxi University of Science and Technology, Ganzhou, 341000, China; d Jiangxi provincial key laboratory of environmental geo-technology and engineering disaster Control, Ganzhou, 341000, China

**Keywords:** Navel orange residue, Waste activated sludge, Pre-treatment, Co-digestion, Volatile fatty acids

## Abstract

In this study, the co-digestion system with Navel orange residues (NOR) and Waste activated sludge (WAS) was established, by pre-treating the NOR and setting different volatile solids (VS) ratios of NOR to WAS to motivate the production of volatile fatty acids (VFA). The pre-treatment method (pH 7 and temperature 70 °C) promoted the release of dissolved organic matter, and the concentration of soluble chemical oxygen demand (SCOD) increased by 45.56% compared with the untreated group (pH 3 and temperature 20 °C). In the co-digestion system, the highest VFA yield (5716.69 mg/L) was obtained at VS ratio of 2. When the VS ratio was increased to 4, the imbalance in proportions of carbon and nitrogen affected VFA production, and the high concentration of essential oils (EO) present in the NOR inhibited the methane production; the cumulative yield of methane gas decreased by 24.10% compared with the yield obtained when the VS ratio was 2. Analysis of microbial community revealed that an increase in the number of VFA-producing microbial populations and the abundance of *Methanobacteria* resulted in the accumulation of acetic acid. This study demonstrated that co-digestion of NOR with WAS improve VFA production, thus realizing the utilization of solid wastes and reducing environmental pollution.

## 1 Introduction

With the rapid development of industrialization and urbanization, the output of waste activated sludge (WAS) in wastewater treatment plants is increasing every year, rendering its treatment cost higher. Anaerobic fermentation of large amount of organic matter present in WAS in wastewater treatment plants produces several valuable compounds and bioenergetic resources; among them, volatile fatty acids (VFA) are valued as they are biodegradable and have many applications, which can be used as a raw material for esters and bioplastic, food or medicated additives, microbial fuel cells, etc. [1,2]. The market price of VFA could reach up to 1500 $/t globally [3]. Therefore, increasing number of studies have focused on anaerobic fermentation of organic wastes such as WAS to produce acid. However, the disadvantages of the sludge used during the single-substrate fermentation process are poor degradability, long digestion period, and poor acid and gas yield [4]; further, the ratio of carbon to nitrogen in the sludge is low, rendering the anaerobic digestion challenging [5]. In recent years, the co-digestion of various organic solid wastes to produce VFA has become one of the hot spots in anaerobic fermentation technology [6,7].

Fruit processing waste is a suitable substrate for anaerobic treatment as it can produce energy, is rich in organic matter, and the volatile solids (VS) present in it account for more than 90% of the total solid content [8]. Every year, about 5,000,000 t of citrus waste residues are produced in China; of which, a small part is used to extract gum and feed additives, and the remaining is randomly discarded or buried [9]. In the past years, a large number of citrus waste residues have not been effectively utilized, which not only leads to serious waste of resources but also causes the spread of mildews and odour, in addition to environmental pollution because of the acidity of citrus wastes [10]. Therefore, researchers have studied biogas production by anaerobic fermentation using citrus waste residue. Li et al. [11] noted out that during the anaerobic fermentation of citrus peels, 1.05 m^3^ of biogas per kg of total solids (TS) was produced, and the methane (CH_4_) content in the biogas was as high as 69.50%. Calabrò and Panzera [12] observed that in the process of ensiling of orange peel waste, microorganisms could use water-soluble carbohydrates for lactic acid fermentation, and the cumulative CH_4_ production of orange peel residue ensiled for 37 d could reach 365 Nml/g VS. Pre-treatment of Citrus residue can improve its anaerobic fermentation efficiency. Xi et al. [13] found that after pre-treatment of citrus residues at 70 °C with 7.5% NaOH (pH 11) for 6 h improved the efficiency of anaerobic fermentation, as the CH_4_ potential after anaerobic fermentation was 258.7 mL/g VS, which was 39.9% higher than the value obtained on fermentation of the control sample.

Many studies have been conducted on anaerobic co-digestion of WAS and fruit processing waste to improve the efficiency of anaerobic fermentation of WAS. Zhao et al. [14] pointed out that the biogas yield was 1.6 and 3.04 times higher than the yields obtained by anaerobic digestion of WAS and citrus dregs alone respectively when citrus dregs and WAS (ratio 1:2, calculated using VS concentrations) were co-digested anaerobically. Hallaji et al. [15] investigated the co-digestion of WAS with mixed fruit waste and cheese whey. The results showed that the activity of protease and cellulase increased by 22 and 9%, and the amount of cumulative CH_4_ yield increased by 31%, compared with the values obtained on digestion of mixed fruit waste and cheese whey separately. However, the anaerobic treatment of WAS and fruit waste was mainly aimed at anaerobic methane production rather than anaerobic acid production, because the efficiency of anaerobic acid production needs to be further improved. In order to increase the VFA yield, the following measures can be taken during the anaerobic fermentation process: 1) Increasing the extent of sludge hydrolysis and the release and biodegradability of dissolved organic matter by providing conditions favourable for acid production [16]. 2) Adopting two or more substrates to co-ferment, increasing the supply of carbon sources to the anaerobic system, diluting the concentration of toxic compounds in the system, balancing the ratio of carbon to nitrogen and improving the buffering capacity of the system [17,18]. 3) Increasing the accumulation of VFA by inhibiting the methanogenic process, which prolongs the duration of the acid production step [19,20]. For the third aspect, the research on inhibition of methanogenesis mainly focuses on the selection of methanogenic inhibitors, that is, the effect of inhibitors on the activity of methanogens and on methane production and acid accumulation.

In recent years, plants or fruits containing essential oils, saponins, tannins and other chemicals, have attracted attention due to their potential methanogenic inhibition [20,21]. The research shows that the essential oil of orange peel can inhibit the anaerobic digestion of methane production. According the result of Zema et al. [22], when citrus waste (concentration of essential oil 88.1 mg/(L‧d); organic load 1.98 g/(L‧d)) was anaerobically fermented, the production of methane was partially inhibited; when the concentration of essential oil was 111.2 mg/(L‧d) and the organic load was 2.5 g/(L‧d), the production of methane was almost completely inhibited. d-limonene is the functional substance present in orange peel essential oil. Previously, the effect of variation in d-limonene concentration on methane production was analyzed by performing anaerobic co-digestion of sludge and orange peels. The results showed that the quantity of methane produced using pre-treated (d-limonene concentration was reduced) tangerine peel wine was 70% higher than that obtained using blank tangerine peel wine (with unchanged d-limonene content) and surplus sludge [23].

Anaerobic co-digestion of navel orange residues (NOR) and WAS (both are sources of biomass energy) will result in the utilization of solid wastes and reduction of the environmental pollution caused by them. The essential oils present in NOR can destroy microbial cell membranes and inhibit methanogenic bacteria [24]. Therefore, this study aimed to provide a technical way to significantly improve the anaerobic acid production performance and stability of the co-digestion system based on NOR and WAS. To address it, the effect of alkali-heat pre-treatment on cracking of NOR was investigated, co-digestion of NOR and WAS with different substrate ratios was performed to analyse the efficiency of acid production and the inhibition of methane production. In addition, the evolution of microbial community under the optimal substrate ratio was analyzed to demonstrate the microbial factors associated with the improved performance and stability.

## 2 Material and methods

### 2.1 Experimental equipment

**Fig. 1:**
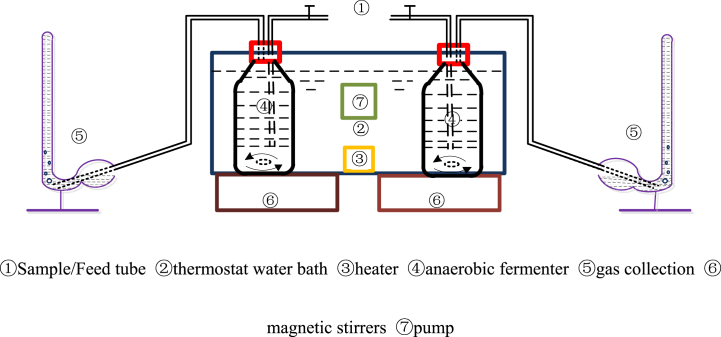
Schematic Diagram showing the anaerobic fermentation equipment.

The anaerobic fermentation reactor was composed of three major parts: the anaerobic fermentation tank, composed of six 1-L anaerobic fermentation bottles (containing 800 mL substrate) placed in a magnetic rotor sealed with rubber plugs; a self-made constant temperature water bath—whose temperature was maintained at 35 ± 1 °C with the help of a heating rod—with its lower part equipped with two magnetic stirrers, which were used to stir the water in the bath every 8 h for 15 min at a speed of 120 rpm to speed up the anaerobic digestion and acid production rates; the gas gathering device, which comprised six 200-mL Smith fermentation tubes containing 2 mol/L NaOH solution. The gas collected in the Smith fermentation tube was mainly methane, and the volume of the gas produced was measured. A schematic diagram of the anaerobic fermentation device is shown in Fig. 1.

### 2.2 Inoculated sludge, WAS and NOR

Waste activated sludge and inoculated sludge were taken from a pond in a wastewater treatment plant and an anaerobic biogas digester of a piggery in Ganzhou City, Jiangxi Province, respectively. The sludge was filtered using a 1 mm mesh-sized sieve and stored in a refrigerator at 4 °C for later use. The Newhall variety of navel oranges planted in Gannan, Jiangxi Province, was used in this experiment. The orange peels were chopped and mixed with a small amount of pulp, crushed using a juicer, mixed with an equal volume of distilled water, and stored in a refrigerator at 4 °C. The physical and chemical properties of inoculated sludge, WAS, and NOR are listed in Table 1.

**Table 1:** Key properties of WAS, inoculated Sludge and NOR.

Parameter	WAS	Inoculated Sludge	NOR
pH	7.02	6.89	3.01
TS (g/L)	30.30	41.20	48.98
VS/TS (%)	45.87	74.51	96.30
TCOD (mg/L)	10033.30	18266.70	45980.00
SCOD (mg/L)	114.00	233.33	24234.13
SC (mg/L)	10.40	22.90	7613.26
SP (mg/L)	0.39	7.35	26.95
TN (mg/L)	343.18	590.45	132.00
TP (mg/L)	23.57	56.58	12.05
VFA (mg/L)	64.14	126.81	72.22

The data in the table was the average of three tests. The parameters of TCOD, SC, SP, TN, and TP represent total chemical oxygen demand, soluble polysaccharide, soluble protein, total nitrogen and total phosphorous, respectively.

### 2.3 Experimental methods

#### 2.3.1 Alkali-heat pre-treatment of NOR

The NOR was divided into 10 parts (100 mL each), which were placed in 150 mL conical flasks. The reactors (flasks containing NOR) were categorized into two sets of five reactors each. The pH of the first set of five reactors was adjusted to 7 and the pH of the second set of reactors was adjusted to 12 using 2 mol/L NaOH. One reactor each from the two sets of reactors was then incubated in a constant temperature water bath at different temperatures (50, 60, 70, 80, and 90 °C) for 60 min. The blank control samples were pre-treated following the same procedure. The heated samples were centrifuged at 8000 rpm for 10 min, and the supernatant was filtered using 0.45 μm pore-sized filter membranes. Finally, the filtrate was used for further analysis.

#### 2.3.2 Anaerobic co-digestion of NOR and WAS

The NOR pre-treated by alkali-heat was mixed with different concentrations of WAS (VS ratios 4.0, 2.0, 1.0, 0.5, and 0.25, named as 1#, 2#, 3#, 4#, and 5#, respectively), and placed in fermentation bottles (720 mL of the solution containing WAS and NOR was mixed with 80 mL of the inoculated sludge). Experiment on the control group (represented by CG; containing only sludge) was performed following the same procedure. To allow anaerobic fermentation to occur, the fermentation bottles were filled with nitrogen, sealed, and subsequently incubated in a water bath maintained at a constant temperature of 35 °C for 15 days. After every 12 h, the water in the bath was stirred at a speed of 120 rpm for 15 min. The gas production was monitored every day, and the VFA produced were measured along with other relevant indicators. The physicochemical properties of the mixtures contained in the fermentation bottles are shown in Table 2.

**Table 2:** Properties of NOR mixed with WAS in different proportions.

Parameter	CK	1#	2#	3#	4#	5#
pH	6.98	6.85	6.83	6.87	6.88	6.91
TS (g/L)	30.48	37.60	33.00	32.50	31.20	31.05
VS/TS (%)	48.27	67.82	62.42	57.54	56.73	51.20
TCOD (mg/L)	11756.64	32506.67	28427.29	23081.54	18973.41	16381.51
SCOD (mg/L)	126.67	10666.67	6533.40	5560.00	3266.67	1700.00
SC (mg/L)	15.75	3732.00	1907.20	1712.00	751.50	445.80
SP (mg/L)	0.55	65.40	46.00	24.75	5.45	4.35
TN (mg/L)	369.21	274.02	287.97	294.53	304.91	346.77
TP (mg/L)	50.08	43.86	51.06	53.26	47.70	53.48
VFA (mg/L)	111.66	1567.46	1541.58	1487.58	1237.79	721.92
EO (mg/L)	0	183.38	113.28	57.60	17.92	0.47

The data in the table was the average of three tests.

### 2.4 Analytical methods

The routine parameters of sludge and NOR were measured using the national standard method of analysis [25]. pH was determined using the glass electrode method, TCOD and SCOD were determined using the fast closed digestion method, TS and VS concentrations were determined using the gravimetric method, TN concentration was determined by alkaline potassium persulfate ultraviolet spectrophotometry, TP concentration was determined by molybdate spectrophotometry, SP concentration was determined using the Coomassie brilliant blue G-250 method, and SC concentration was determined using the phenol-sulfuric acid method. EO yield was estimated using the distillation–titration method [26]. Concentrations of dissolved extracellular polymeric substances (EPS), loosely bound EPS, and tightly bound EPS were determined using the method of extraction reported by Zhao [27]. The particle size of NOR was detected by the wet method using a Mastersizer 3000 laser particle sizer, and the morphology of NOR was examined using a scanning electron microscope.

The concentration of VFA was determined by gas chromatography (Agilent 7890B, USA) equipped with hydrogen flame ion detector (FID) and DB-FFAP capillary column (the specification was 30 m × 0.25 mm × 0.25 μm, the carrier gas was nitrogen, and the flow rate was 30 mL/min). FID detector temperature was 280 °C, injection inlet temperature was 250 °C, split ratio was 20:1, and single injection volume was 1.0 μL. The temperature rise procedure is as follows: the initial temperature is 60 °C, stay for 5 min, and then rise to 220 °C with the procedure of 20 °C/min, and stay for 5 min. VFA is mainly composed of six organic acids, namely acetic acid, propionic acid, n-butyric acid, isobutyric acid, n-valeric acid and isovaleric acid.

The concentrations of coenzyme F_420_ (affects anaerobic methanation) and acetate kinase (AK; affects acetic acid production during anaerobic fermentation) were determined using ultraviolet (UV) spectrophotometry. The microbial community structure in the anaerobic system was determined using the Illumina 16S rRNA sequencing platform. First, the Qubit3.0 DNA detection kit was used to quantify the genomic DNA. For the first round of PCR, the primers that were used for amplification of the V3–V4 16S rRNA region were procured from Illumina. For the second round of PCR, Illumina bridge PCR compatible primers were used.

### 2.5 Calculation

The degree of disintegration (DD) of NOR was calculated to determine the extent of cracking of NOR. The calculation method was performed according to the following equation [28]:

$$\text{DD}=\frac{{SCOD}_{a}-{SCOD}_{0}}{{TCOD}_{0}-{SCOD}_{0}}\times 100\;\%$$

where *SCOD*_*a*_ is the concentration of dissolved COD in pre-treated NOR; *SCOD*_*0*_ is the concentration of dissolved COD in untreated NOR; and *TCOD*_*0*_ is the total concentration of COD in NOR.

## 3 Results and discussion

### 3.1 Effect of alkali-heat pre-treatment on cracking of NOR

#### 3.1.1 Effect of pre-treatment on soluble organics and total solids

**Fig. 2:**
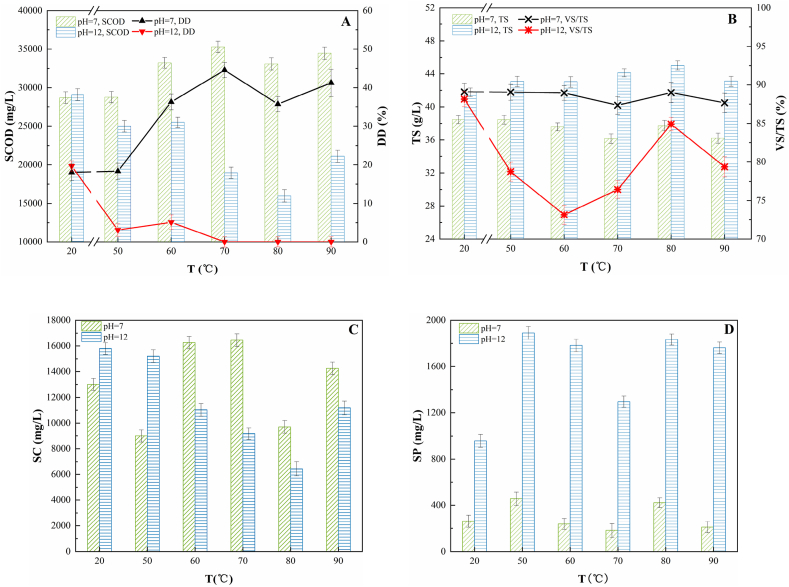
Influence of alkali-heat pretreatment on the concentration of SCOD and TS. (A) SCOD and DD; (B) TS and VS/TS; (C) SC; and (D) SP.

Changes in the concentrations of SCOD, TS, and other indicators were observed in each of the analyzed samples after the NOR was cracked by alkali-heat pre-treatment (Fig. 2). Marked differences were observed in the SCOD concentrations and the DD values of pre-treated samples whose pH had been adjusted to 7.0 and 12.0 (Fig. 2A). At pH 7.0, with the increase in temperature, the SCOD concentrations and DD values of the pre-treated samples first increased and then stabilised. The cracking rate was the highest at 70 °C, and the SCOD concentrations and DD values increased by 22.91 and 26.55%, respectively, compared with the values observed for the control samples (pH = 7, temperature [t] = 20 °C); the values increased by 45.56 and 44.58%, respectively, for the pre-treated samples compared with the values obtained for the untreated samples (pH = 3, t = 20 °C). At pH 12.0, the SCOD concentrations and the DD values of the pre-treated samples were significantly lower than those of the control samples (pH = 12, t = 20 °C). At higher temperatures, the extent of cracking reduced. Further, the VS/TS ratio had an opposite relationship with the degree of cracking of NOR, that is, the smaller the VS/TS ratio, the higher the degree of cracking.

At pH 7.0, the extent of degradation of VS in the pre-treated samples was observed to be the highest at 70 °C. The VS/TS ratios in the pre-treated samples were 1.72% lower than the values obtained for the control group (pH = 7, t = 20 °C) and 9.05% lower than the values obtained for the untreated group (Fig. 2B). At pH 12.0, when the temperatures were increased, the VS/TS values and the SCOD concentrations of the pre-treated samples exhibited pronounced fluctuations, and the values were lower than those obtained for the corresponding control groups (pH = 12, t = 20 °C). To sum up, alkali-heat pre-treatment resulted in a decrease in the VS concentration. However, high temperatures and alkaline conditions enhanced the formation of macromolecular polymers, which hindered the release of SCOD [29].

At pH 7, the SC concentrations in the pre-treated samples fluctuated greatly with the increase in temperature (Fig. 2C). The SC concentration was the highest at 70 °C, which was 26.46% higher than the values obtained for the corresponding control group and 1.15 times higher than the values obtained for the untreated group. At pH 12, the pattern of change in SC concentrations was similar to that of SCOD values, except at 90 °C, where the SC concentrations in the pre-treated samples decreased with the increase in temperature. The results obtained in this experiment are similar to those of Jard et al. Jard et al. [30] observed that when red algae were pre-treated with 0.04 g NaOH/g TS at 70 and 85 °C, the SC concentration at 85 °C was higher than that at 70 °C, but lower than that in the untreated group. The reason could be that under the strong alkaline conditions, the Maillard effect occurred, resulting in the combination of SCs with proteins because of the increase in temperature [31]. At pH 7 and 12 (Fig. 2D), the SP concentrations in the pre-treated samples showed varying degrees of fluctuation with the increase in temperature, but the trend of change was similar to that observed for SC concentrations. The extent of release of SP was the highest at 50 °C. At pH 7 and temperature 50 °C, the SP concentrations in the pre-treated samples increased by 74.19% compared with the values obtained for the corresponding control groups. The SP concentrations in the pre-treated samples were 15.94 times higher than the SP concentrations in the untreated samples. At pH 12 and temperature 50 °C, the SP concentrations in the pre-treated samples increased by 97.23% compared with the concentrations in the corresponding control groups, and these values were 66 times higher than those observed for the untreated samples.

#### 3.1.2 Effect of pre-treatment on particle size and morphology of NOR

**Fig. 3:**
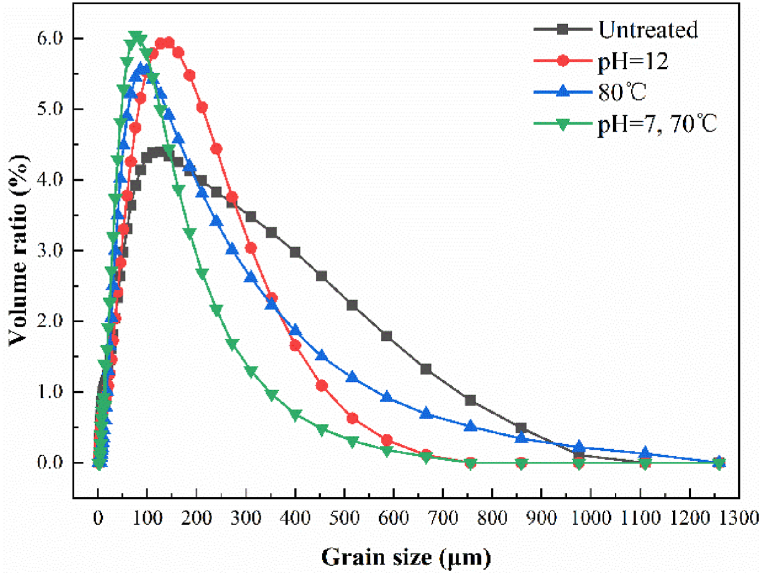
Particle size analysis of NOR.

The average size of NOR particles in the untreated samples was 125.5 μm, while the average sizes of NOR particles in the samples that underwent pre-treatment at pH 12, thermal treatment at 80 °C, and a combination of alkali and heat treatment at pH 7 and temperature 70 °C were 101, 91, and 58.3 μm, respectively (Fig. 3). The average size of NOR particles in samples pre-treated at pH 7 and 70 °C was smaller than that in samples pre-treated with alkali or heat alone. Compared with the average size of NOR particles in the untreated samples, the average size in samples pre-treated at pH 7 and 70 °C decreased by 46%. The observed size of NOR in the pre-treated samples was lower than the size of mechanically pre-treated orange peels that was reported by Yi Tian et al. [32]. These findings suggest that combined pre-treatment using alkali and heat (pH 7 and 70 °C) results in a decrease in the particle size of NOR, which is then effectively degraded by microorganisms during the process of anaerobic fermentation.

**Fig. 4:**
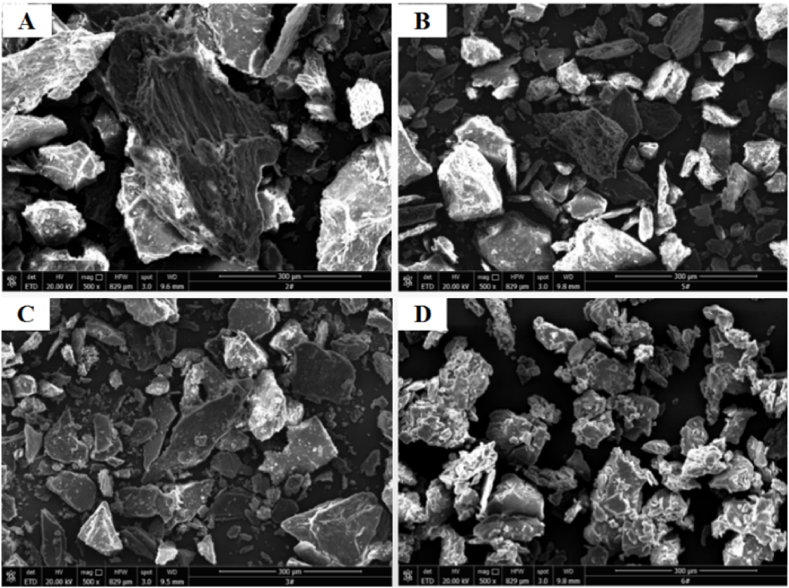
Morphological analysis of NOR. (A) untreated; (B) pH = 12; (C) 80 °C; and (D) pH = 7, 70 °C.

Morphological analysis of NOR was performed using a scanning electron microscope (Fig. 4). In the untreated samples, the NOR particles’ surface was uneven. The NOR particles were found to be irregular with large block-shaped dense structures, which hindered the degradation of lignocellulosic materials by microorganisms [33]. After alkali treatment at pH 12, thermal treatment at 80 °C, and combined pre-treatment at pH 7 and 70 °C, the morphology of NOR changed to different degrees; the particle size of NOR decreased and its structure became fragmented. Most of the small particles were evenly distributed on the surface of the conductive adhesive material. The combined pre-treatment at pH 7 and 70 °C enhanced the breakdown of large NOR particles into smaller particles. The surfaces of the large particles were cracked and fragmented because of fracture and expansion of some functional groups in the lignocellulose of NOR and reduction of the phenolic acids [34]. These conditions were conducive to the attachment of microorganisms. Thus, the rate of biodegradation of NOR increased.

### 3.2 Analysis of VFA produced by anaerobic co-digestion of NOR and WAS

#### 3.2.1 Changes in VFA yield and its composition

**Fig. 5:**
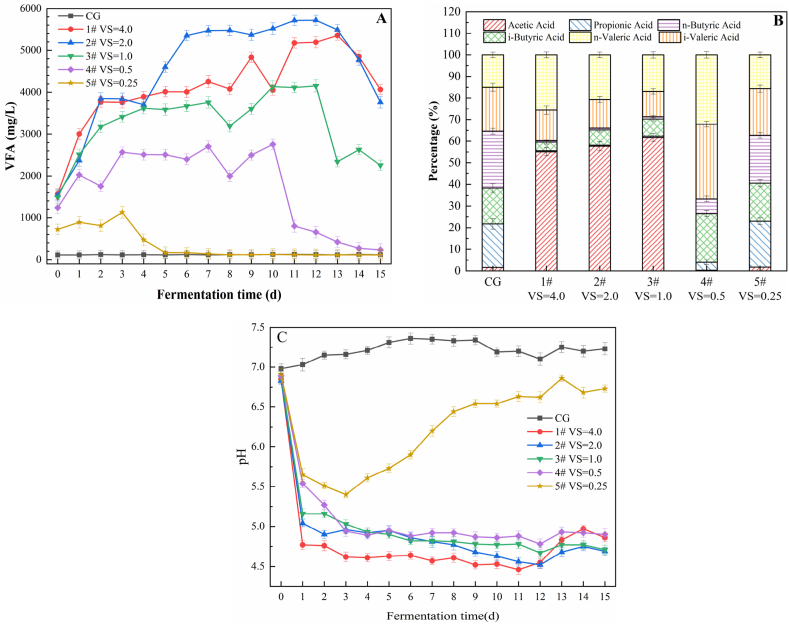
Changes in (A)VFA yield, (B)VFA composition and (C) pH.

According to the VS ratio, the pre-treated NOR (pH 7 and 70 °C) was mixed with WAS in different proportions (Table 2) for anaerobic co-digestion. Changes in VFA yield and its composition during anaerobic co-digestion are shown in Fig. 5. The VFA yields obtained from the experimental samples were higher than that of the CG. In general, the yield increased initially and then decreased during the reaction (Fig. 5A). The reason was that in the early stages of the anaerobic reaction, sufficient quantity of the organic matter was available for the microorganisms to produce VFA by fermentation; in the later stages of fermentation, the quantity of organic matter did not meet the metabolic requirements of the anaerobic acidogenic bacteria. In addition, the methanogens began to consume acetic acid to produce methane. On day 11 of the anaerobic reaction, the VFA yield of experiment 2# was the highest at 5716.69 mg/L, which was 3.7 times greater than the initial value. Except that of experiment 1#, the VFA yield of the other groups showed a gradual increase with the increase in the VS ratio of the solution containing NOR and WAS. The reason was that the proportion of organic matter in the anaerobic system increased because of the presence of NOR, which enhanced the generation of VFA. The carbon/nitrogen (C/N) ratio is one of the most important reference indices for the evaluation of anaerobic digestion [35]. The VFA yield in experiment 1# was lower than that in experiment 2#, possibly because the C/N ratio in experiment 1# (about 42.3) was higher than that in experiment 2# (about 28.2), which caused imbalance in the proportion of the available nutrients; this situation was not conducive to the formation of VFA. Previous studies have shown that the optimal ratio of C/N in an anaerobic digestion system is between 20 and 30, and C/N ratios higher or lower than this range of values might damage microbial cell functioning and affect anaerobic digestion [36]. In addition, the VFA content was closely related to the pH value; high VFA yields were obtained under low pH conditions (Fig. 5C). The pH values of experiments 1#–4# rapidly decreased in the initial stages of the reaction, and then stabilised at about pH 5; the pH values of experiment 5# and CG ranged between 6.7 and 7.2 in the stable state.

The relationship between the C/N ratios of the substrate and the microorganisms’ efficiency of digestion is reflected by the change in the composition of VFA. Navel orange residue contains a large amount of saccharide, while the soluble protein content is relatively low (Table 2). The proportion of acetic acid was the highest in experiments 1#, 2#, and 3#, accounting for 55.12, 57.67, and 61.73% of the total VFA, respectively; the proportion of propionic acid and isovaleric acid was the lowest, and the sum of the percentages of these two acids was less than 5% in each group (Fig. 5B). The results showed that the highest degree of acetic acid fermentation occurred in experiments 1#, 2#, and 3#, and the addition of an appropriate amount of NOR to the anaerobic co-digestion system enhanced the generation of acetic acid. Butyric acid and valeric acid fermentation was observed in experiment 4#, both of which accounted for 56.78% of the total VFA. Experiment 5# exhibited mixed acid fermentation, and the acetic acid yield was the lowest in this experiment, which may have been caused by the consumption of acetic acid by the methanogens in the fermentation tank. These results indicate that the C/N ratio affected the metabolic processes in the microorganisms that carried out anaerobic fermentation. Thus, they performed different types of fermentation reactions. High C/N ratios resulted in a decrease in the length of the carbon chains of the VFA. These results are consistent with the findings of Agler et al. [37].

#### 3.2.2 Changes in concentrations of soluble organics and EPS

**Fig. 6:**
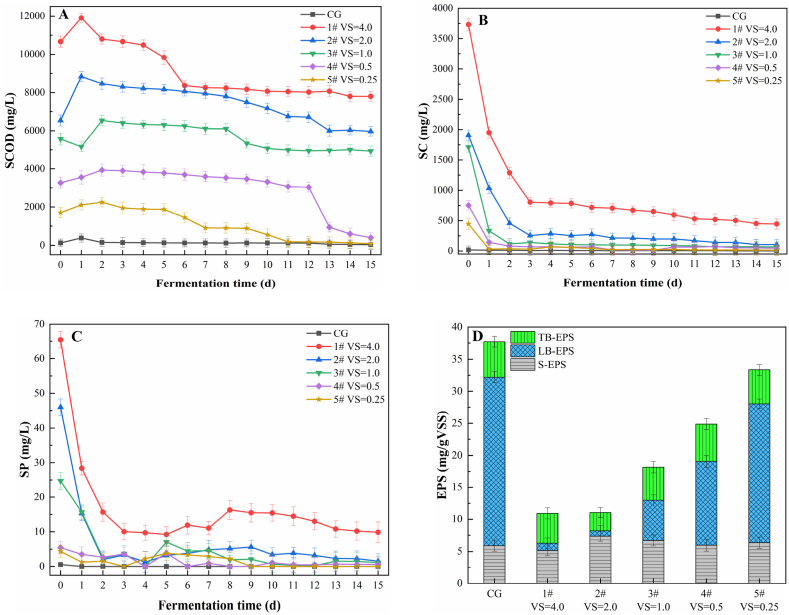
Changes in concentrations of soluble organic matter and EPS. (A) SCOD; (B) SC; (C) SP; and (D) EPS.

The changes in concentrations of SCOD, SC, SP, and sludge EPSs are shown in Fig. 6. The COD content reflects the degree of hydrolysis of the particulate organic matter during anaerobic fermentation. The value of SCOD was observed to be the highest on day 2 of anaerobic fermentation (Fig. 6A), which indicated that insoluble organic matter in the mixed substrates was hydrolysed into soluble organic matter by microbial action in the initial stages of the anaerobic reaction. In the mid- and late-anaerobic stages, the degree of hydrolysis reduced and the microorganisms began to use the organic matter in large quantities. The SCOD concentrations in experiments 1#–5# showed a gradual downward trend; at the end of the anaerobic fermentation, the SCOD concentrations in the experiments 1#–5# were 205.26, 157.02, 129.82, 10.53, and 2.19 times higher than the concentrations in the control samples, respectively. The SC and SP values predominantly contributed to enhancing the initial SCOD concentrations. The SC and SP contents in all the experimental groups decreased rapidly in the first three days of the anaerobic reaction, and then the rate of decrease slowed down and became stable in the final stages of the reaction (Fig. 6B and C). The SC and SP constitute easily degradable soluble organic matter. Thus, these are preferentially utilized by anaerobic microorganisms, which might have resulted in the rapid decrease in their concentrations in the initial stages. In the mid- and late-anaerobic stages, the SCOD, SC, and SP concentrations became stable, indicating that the polysaccharides were degraded into glucose and the proteins were degraded into substrates such as amino acids.

Considering the difficulties that microorganisms face in utilizing different types of molecules, the EPS content of sludge can be categorized into tight binding-EPS (TB-EPS), loose binding-EPS (LB-EPS), and soluble-EPS (S-EPS); S-EPS can be dissolved in aqueous solutions and is most easily utilized by anaerobic microorganisms, while LB-EPS surrounds the sludge cells and is flocculent, so it is difficult to use it directly, and TB-EPS is closely combined with the sludge cells and is the most difficult to use. In the process of anaerobic fermentation, the hydrolysis of granular proteins and carbohydrates (the main components of EPS) is a necessary step in the synthesis of VFA (27). At the end of anaerobic co-digestion, the total EPS concentrations of experiments 1#–5# were lower than those of the control samples. The EPS concentrations decreased with increase in the VS ratio of NOR to WAS in the experimental samples (Fig. 6D). The changes in the ratios of TB-EPS and S-EPS in the experimental groups were relatively negligible, ranging from 0.4 to 0.9; while the ratios of LB-EPS to S-EPS changed markedly, ranging from 0.1 to 4.5. The S-EPS concentration in experiment 2# was the highest, that is, the conversion rates of TB-EPS and LB-EPS to S-EPS were the highest, indicating that the introduction of NOR to the co-digestion system enhanced the dissolution of soluble organic matter; the reason may be that the EO in NOR promoted the solubilization of the mixed substrates, thus increasing the quantity of degradable organic matter that was available for utilization by the anaerobic acidogenic bacteria.

#### 3.2.3 Change in methane production and microbial enzyme activity

**Fig. 7:**
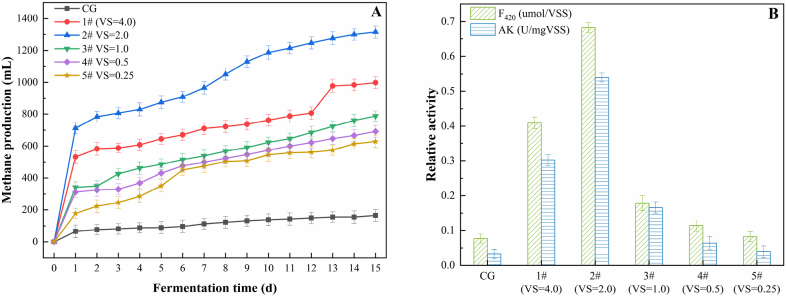
Variation in (A) methane yield and (B) microbial enzyme activity.

The effect of variation in substrate ratios on the cumulative methane yield during anaerobic co-digestion is shown in Fig. 7A. The cumulative methane yields of the experimental groups were noticeably higher than the yield obtained from the CG, indicating that increase in the dosage of NOR enhanced the anaerobic fermentation efficiency. Except experiment 1#, the cumulative methane yields of experiments 2#–5# decreased with the decrease in the VS ratio of NOR to WAS in the samples. The cumulative methane yield of experiment 2# was the highest, which was 1.15 and 7.96 times higher than the yields obtained from experiment 5# and CG, respectively. Therefore, addition of the appropriate amount of NOR to the anaerobic fermentation system increased the quantity of organic matter, which favoured methane accumulation. However, when present in excess, NOR might inhibit the methanogenic process to some extent. Previous studies have shown that the essential oil contained in NOR inhibits methane production [38]. Navel orange peel is rich in essential oil, and the proportion of d-limonene in the essential oil is 92–93% [39]. d-limonene is a safe and harmless antibacterial compound. If a large amount of limonene gets accumulated on the surface of microorganisms, it could either lead to a decrease in the quantity of unsaturated fatty acids in the microbial cell membranes or change/destroy the membrane components. Moreover, it could modify proton dynamics in the cell, thus resulting in an antibacterial effect. Rosangela et al. [40] showed that at sublethal concentrations, limonene caused marked changes in the cell membranes of thermophilic octococcus methanogens, thus demonstrating the antibacterial effect of limonene. The EO concentration in experiment 1# was 1.6 times higher than that in experiment 2#, and the cumulative methane yield of experiment 1# was 24.10% lower than that of experiment 2#, suggesting that methanogenesis was inhibited in experiment 1#. The results of this experiment were similar to the findings of a previous study, in which plant essential oil was added to a sample that subsequently underwent *in vitro* rumen fermentation [41]; that is, at an optimal concentration of essential oil, the rate of anaerobic fermentation was enhanced and the acid yield was increased, but when the concentration of essential oil was high, methanogenesis was inhibited to some extent.

Some enzymes involved in the anaerobic fermentation pathway play a key role in regulating substrate metabolism and formation of the final products, among which, AK is an important indicator reflecting the performance of acetic acid production. The higher the concentration of AK, the stronger the activity exhibited by acetic acid-producing bacteria [42]. The concentration of coenzyme F_420_ influences the activity of anaerobic methanogens. The higher the concentration of coenzyme F_420_, the stronger the activity of methanogens [43]. Fig. 7B shows the changes in concentrations of coenzyme F_420_ and AK in the anaerobic fermentation tanks. The levels of activities shown by the two enzymes in the experimental groups were higher than the levels observed for the CG. The activities of the two enzymes were the highest in experiment 2#; the values were 18 and 8.5 times higher than that of the CG, respectively. However, the activity levels of these two enzymes in experiment 1# were 3 and 5 times lower than that in experiment 2#, respectively, indicating that the high EO content in experiment 1# exerted an inhibitory effect on the processes of acid and methane production. The enzyme activities in the experimental groups were positively correlated with the yields of VFA and methane, which showed that the introduction of an optimal amount of NOR could increase the overall enzyme activity of the anaerobic digestion system, strengthen the acid production and fermentation processes, and enhance the stability of the anaerobic system.

#### 3.2.4 Changes in microbial community structure

**Fig. 8:**
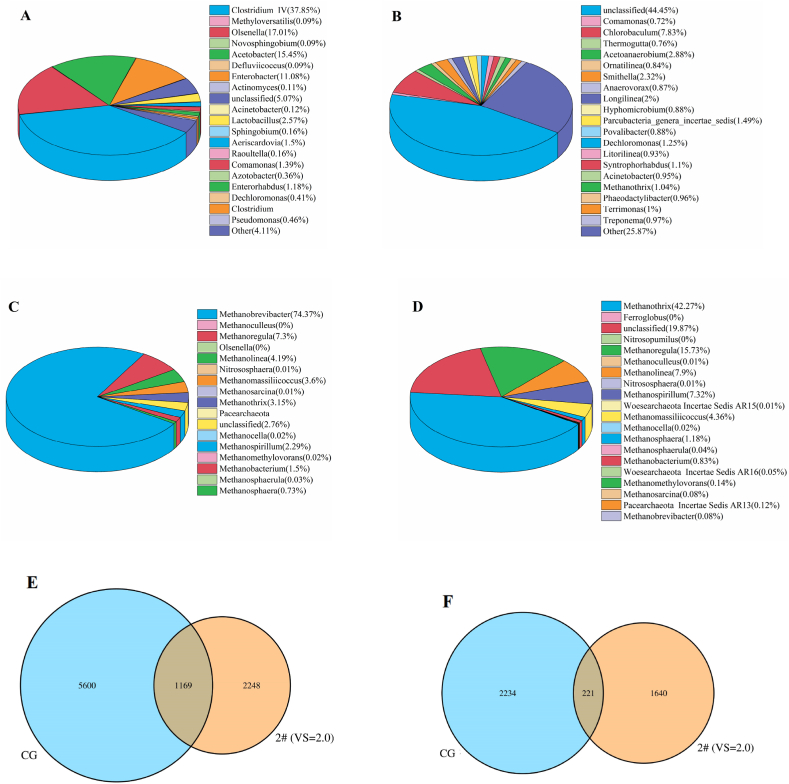
Changes in the microbial community structure. (A/C) bacterial and archaea population distribution in experiment 2#; (B/D) bacterial and archaea population distribution in CG; (E/F) Venn diagram of bacterial and archaea population distribution.

The efficiency of anaerobic digestion is closely related to the microbial community structure, and different substrate ratios may change the anaerobic digestion conditions, thus affecting microbial diversity [44]. The microbial community structures in CG and experiment 2# were compared and analyzed using the metagenome classification and sequencing technology (Fig. 8).

Fig. 8 (A, B) shows the changes in the bacterial populations in experiment 2# and CG, respectively. The dominant bacterial phyla in experiment 2# were *Proteobacteria*, *Firmicutes*, *Actinobacteria*, and *Bacteroidetes*, and their abundance values were 33.47, 42.46, 20.44, and 1.22%, respectively. Compared with the bacteria observed in CG, many *Actinobacteria* were present in experiment 2#. The relative abundance values of *Proteobacteria* and *Firmicutes* increased by 25.73% and 3.64 times, respectively. However, the abundance of *Bacteroidetes* decreased by 93.65%. Earlier studies have shown that many *Firmicutes* and *Proteobacteria* can metabolize different substrates to produce acetic acid during anaerobic digestion [45]. Some *Actinomycetes* can produce extracellular hydrolases such as proteases, lipases, and polysaccharide hydrolases, whose action increases the quantity of degradable organic matter available for acid and gas production in the later stages of anaerobic fermentation [46]. Therefore, the increase in populations of *Proteobacteria*, *Firmicutes*, and *Actinobacteria* in experiment 2# enhanced VFA yield. At the genus level, the dominant bacteria in experiment 2# were *Clostridium* cluster IV (37.85%), *Olsenella* (17.01%), *Acetobacter* (15.45%), and *Enterobacter* (11.08%). *Clostridium* cluster IV belongs to the class Clostridia of *Firmicutes*, *Olsenella* belongs to *Actinomycetes*, and *Acetobacter* and *Enterobacter* belong to *Proteobacteria*. Compared with the abundance of the bacterial population in CG, the abundance value of the bacterial population in experiment 2# decreased by 59.86% (Fiure 8E), while the population of VFA-producing microorganisms notably increased. This was one of the reasons for the increase in VFA yield in the anaerobic co-digestion system containing NOR and WAS.

Fig. 8 (C, D) shows the changes in the populations of archaea in experiment 2# and CG, respectively. At the phylum level, the dominant archaea in experiment 2# and CG were *Euryarchaeota*. Compared with the abundance values of archaea populations in CG, the relative abundance value of *Methanobacteria* in experiment 2# increased by 35.49 times, while the relative abundance value of *Methanomicrobia* decreased by 76.10%. Difference in the relative abundance values of *Methanomassiliicoccales* was negligible. At the genus level, the dominant methanogen in CG was *Methanothrix* (belongs to *Methanomicrobia*) with a relative abundance value of 42.27%. *Methanothrix* mainly uses acetic acid to produce methane. The dominant methanogen in experiment 2# was *Methanobrevibacter* (belongs to *Methanobacteria*) with a relative abundance value of 74.37%. *Methanobrevibacter* generally does not consume acetic acid; however, it uses H_2_, formate, and other electron donors to reduce CO_2_ in order to produce CH_4_. After the addition of NOR to the experimental sample, the relative abundance value of *Methanobrevibacter* significantly increased than that in the control group. While the total number of archaea decreased by 26.59%, 221 strains observed in experiment 2# and CG were identical (Fig. 8F). In addition, the abundance of *Methanobacteria* increased significantly, which indicated that the addition of NOR into WAS created a habitable environment for *Methanobacteria*, resulting in *Methanobacteria* becoming the dominant microbial population. In summary, the addition of NOR in the co-fermentation system promoted the shift of methane generation pathway from acetyl decomposition pathway to hydrogenated nutrition pathway, which increased the abundance of methanogenic archaea utilizing H_2_ and CO_2_, while decreased the abundance of methanogenic archaea utilizing acetic acid, thereby reducing the consumption of acetic acid and promoting the accumulation of VFA.

## 4 Conclusion

This study revealed that the combined pre-treatment of NOR coupled the addition of NOR was an effective method to increase VFAs production during anaerobic fermentation of WAS. The combined pre-treatment at pH 7 and 70 °C enhanced the release of SCOD from NOR, which was conductive to improve the yield of VFA in the co-digestion system. Adding proper amount of NOR in the co-digestion system increased VFA and methane production. However, excessive addition of NOR decreased the VFA yield due to the imbalance in proportions of carbon and nitrogen, besides, the high concentrations of essential oil inhibited the methane yield. The optimum VS ratio of NOR to WAS in the co-digestion system was determined to be 2 considering the results of the analysis of VFA production, enzymatic activity, and microbial community characteristics.

## Author contribution statement

Shan-Yan Dong: conceived and designed the experiments; analyzed and interpreted the data; contributed reagents, materials, analysis tools or data; wrote the paper.

Jin-Cai Luo: performed the experiments; analyzed and interpreted the data; contributed reagents, materials, analysis tools or data; wrote the paper.

Gang Chen: conceived and designed the experiments; performed the experiments; analyzed and interpreted the data.

Shuai Tian; Xiang-Zhe Xiao: contributed reagents, materials, analysis tools or data.

Yi-Chun Zhu: conceived and designed the experiments; analyzed and interpreted the data.

## Data availability statement

Data will be made available on request.

## Declaration of competing interest

The authors declare that they have no known competing financial interests or personal relationships that could have appeared to influence the work reported in this paper.
